# Patterns in Microbial Assemblages Exported From the Meltwater of Arctic and Sub-Arctic Glaciers

**DOI:** 10.3389/fmicb.2020.00669

**Published:** 2020-04-15

**Authors:** Tyler J. Kohler, Petra Vinšová, Lukáš Falteisek, Jakub D. Žárský, Jacob C. Yde, Jade E. Hatton, Jon R. Hawkings, Guillaume Lamarche-Gagnon, Eran Hood, Karen A. Cameron, Marek Stibal

**Affiliations:** ^1^Department of Ecology, Faculty of Science, Charles University, Prague, Czechia; ^2^Stream Biofilm and Ecosystem Research Laboratory, School of Architecture, Civil and Environmental Engineering, École Polytechnique Fédérale de Lausanne, Lausanne, Switzerland; ^3^Department of Environmental Sciences, Western Norway University of Applied Sciences, Sogndal, Norway; ^4^School of Earth Sciences, University of Bristol, Bristol, United Kingdom; ^5^National High Magnetic Field Laboratory, Department of Earth, Ocean & Atmospheric Science, Florida State University, Tallahassee, FL, United States; ^6^GFZ German Research Centre for Geosciences, Potsdam, Germany; ^7^Department of Natural Sciences, University of Alaska Southeast, Juneau, AK, United States; ^8^Institute of Biological, Environmental and Rural Sciences, Faculty of Earth and Life Sciences, Aberystwyth University, Aberystwyth, United Kingdom

**Keywords:** glacial runoff, 16S rRNA gene, polar stream, biogeography, cryosphere, hydrology

## Abstract

Meltwater streams connect the glacial cryosphere with downstream ecosystems. Dissolved and particulate matter exported from glacial ecosystems originates from contrasting supraglacial and subglacial environments, and exported microbial cells have the potential to serve as ecological and hydrological indicators for glacial ecosystem processes. Here, we compare exported microbial assemblages from the meltwater of 24 glaciers from six (sub)Arctic regions – the southwestern Greenland Ice Sheet, Qeqertarsuaq (Disko Island) in west Greenland, Iceland, Svalbard, western Norway, and southeast Alaska – differing in their lithology, catchment size, and climatic characteristics, to investigate spatial and environmental factors structuring exported meltwater assemblages. We found that 16S rRNA gene sequences of all samples were dominated by the phyla Proteobacteria, Bacteroidetes, and Actinobacteria, with Verrucomicrobia also common in Greenland localities. Clustered OTUs were largely composed of aerobic and anaerobic heterotrophs capable of degrading a wide variety of carbon substrates. A small number of OTUs dominated all assemblages, with the most abundant being from the genera *Polaromonas*, *Methylophilus*, and *Nitrotoga.* However, 16–32% of a region’s OTUs were unique to that region, and rare taxa revealed unique metabolic potentials and reflected differences between regions, such as the elevated relative abundances of sulfur oxidizers *Sulfuricurvum* sp. and *Thiobacillus* sp. at Svalbard sites. Meltwater alpha diversity showed a pronounced decrease with increasing latitude, and multivariate analyses of assemblages revealed significant regional clusters. Distance-based redundancy and correlation analyses further resolved associations between whole assemblages and individual OTUs with variables primarily corresponding with the sampled regions. Interestingly, some OTUs indicating specific metabolic processes were not strongly associated with corresponding meltwater characteristics (e.g., nitrification and inorganic nitrogen concentrations). Thus, while exported assemblage structure appears regionally specific, and probably reflects differences in dominant hydrological flowpaths, OTUs can also serve as indicators for more localized microbially mediated processes not captured by the traditional characterization of bulk meltwater hydrochemistry. These results collectively promote a better understanding of microbial distributions across the Arctic, as well as linkages between the terrestrial cryosphere habitats and downstream ecosystems.

## Introduction

Glacier meltwater streams connect discrete cryosphere habitats with downstream freshwater and marine ecosystems across the Northern Hemisphere (e.g., Hood et al., 2009; O’Neel et al., 2015; Milner et al., 2017). In addition to exporting freshwater, glaciers and ice sheets also subsidize microbial productivity and respiration through the downstream delivery of particulate and dissolved material such as carbon (Bhatia et al., 2013a; Lawson et al., 2014; Kohler et al., 2017), macro- and micronutrients (Bhatia et al., 2013b; Hawkings et al., 2015; Dubnick et al., 2017a), and other weathering products (Hawkings et al., 2017; Hatton et al., 2019a; Stachnik et al., 2019). While recent progress has been made in determining factors that control the magnitude of these biogeochemical fluxes, important clues into solute generation and the operation of the subglacial drainage system may be uncovered through the investigation of more qualitative characteristics of these exports. For example, past work has successfully shown that chemical signatures of dissolved organic matter (Hood et al., 2009; Lawson et al., 2014; Dubnick et al., 2017b) and elemental isotopes (Kohler et al., 2017; Hatton et al., 2019b) are related to the hydrological and lithological characteristics of the glacial environment.

One potentially useful, yet under-utilized, tool for investigating hydrological and biogeochemical weathering processes are the diverse microbial cells collected and exported by meltwater from the glacial ecosystem. For example, subglacial microbes are found at the intersection of the glacier and the underlying bedrock, and are functionally diverse, having been shown to utilize a myriad of metabolic pathways operating over a spectrum of redox conditions (Boyd et al., 2010, 2011, 2014; Stibal et al., 2012a, c; Hamilton et al., 2013; Dieser et al., 2014), which may enable them to influence a host of weathering reactions and biogeochemical transformations (Sharp et al., 1999; Mitchell et al., 2013; Montross et al., 2013; Lamarche-Gagnon et al., 2019). Yet, due to their physical inaccessibility, these habitats are notoriously difficult to investigate, and much of our knowledge of these habitats at present comes from discrete samples taken from marginal areas (e.g., Boyd et al., 2011; Žárský et al., 2018). On the other hand, supraglacial (surface ice) microbial communities, which are comparatively straightforward to access, can include all three domains of life (Anesio et al., 2017), and include oxygenic, phototrophic and carbon-fixing taxa, with Cyanobacteria specifically playing an integral part in forming the matrix of cryoconite found in depressions on the glacier surface (Langford et al., 2010; Cook et al., 2016; Gokul et al., 2019).

Meltwater generated on glacier surfaces collects into supraglacial streams and lakes that eventually drain into moulins and crevasses to enter the subglacial hydrological system (Irvine-Fynn et al., 2011; Hotaling et al., 2017b). Within the subglacial environment, waters may be routed through lower residence time efficient/channelized drainage systems (analogous to subglacial ‘stream channels’), or through a longer residence time distributed system, which may be more analogous to the saturated sediments of rivers (Tranter et al., 1996; Hubbard and Nienow, 1997; Irvine-Fynn et al., 2011). No matter the path, meltwater entrains debris and microbial cells *en route*, and is evacuated from the glacier terminus to form proglacial streams. Thus, a wealth of information on the physical/chemical characteristics and drainage pathways of a given drainage network can be obtained by analyzing the cells suspended in meltwater. Given recent advances in sequencing technologies and bioinformatics, these data have great potential to augment traditional physical and chemical clues for inferring hydrologic patterns and biogeochemical processes among diverse glacial habitats.

The physical characteristics (size and shape) and geographic location of glaciers (latitude, elevation, and aspect) promote differences in seasonal melt patterns and associated hydrological ‘plumbing,’ providing varying levels of meltwater exposure to subglacial habitats (Tranter et al., 1996; Wadham et al., 2010). Subglacial environments themselves are likely heterogeneous within systems (Graly et al., 2014) and across space and time because of differences in hydrologic regime (Tranter et al., 2005), underlying lithology (Mitchell et al., 2013), and organic matter reserves (Stibal et al., 2012c), all of which may dictate possible metabolic pathways and energy sources for microbes. Similarly, microbes inhabiting the supraglacial system can also differ spatially due to differences in dispersal, climate conditions, and allochthonous inputs (Stibal et al., 2012b; Cameron et al., 2016). Therefore, regionally specific assemblages may emanate from glacial rivers across the Arctic and sub-Arctic.

While temporal (Sheik et al., 2015; Dubnick et al., 2017a) and catchment-scale (Hauptmann et al., 2016; Cameron et al., 2017; Žárský et al., 2018) studies on cell export have been previously performed from a limited number of glacier streams, there are currently no studies that have made comparisons among major geographic regions. Thus, in this work, we ask two main questions: (1) How do exported meltwater assemblages compare among disparate, high latitude regions, and (2) can a combination of physical and chemical characteristics be used to explain the exported assemblage structure and provide clues into their origins? To test these questions, we collected and analyzed meltwater samples from glaciers in six major (sub)Arctic regions differing in climate, glacier size, and bedrock lithology. We hypothesized that individual geographic regions should export unique microbial assemblages due to collective differences in latitude, climate, and geology. Furthermore, we predicted that physico-chemical variables commonly used to infer hydrological patterns would be useful in predicting likely sources of microbial cells from the supra- and subglacial environments.

## Materials and Methods

### Study Sites

**FIGURE 1:**
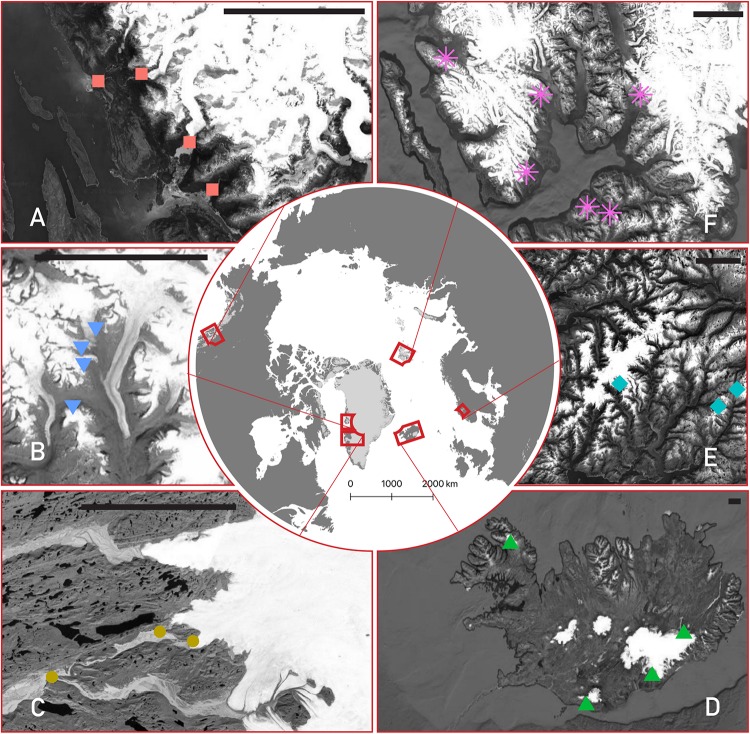
Map of the six studied regions, with detailed insets for each showing the location of each glacier stream sampled. Counterclockwise from top-left: **(A)** southeast Alaska (red squares), **(B)** Qeqertarsuaq (Disko Island, blue upside-down triangles), **(C)** the Greenland Ice Sheet (gold circles), **(D)** Iceland (green triangles), **(E)** Norway (turquoise diamonds), and **(F)** Svalbard (purple asterisks). The scale bar in each panel represents 25 km. Background images are from Landsat/Copernicus and the United States Geological Survey, taken via Google and compiled with QGIS.

Meltwater samples were collected from 24 glaciers over six different Arctic and sub-Arctic regions over the 2015–2017 summers (Figure 1). A full list of their characteristics is given in Table 1. All sites were sampled as close to the glacier terminus as safely possible (most within ∼10 m), with exceptions noted below. Briefly, four streams were sampled from the Kuannersuit Valley, located in central Qeqertarsuaq (Disko Island), west Greenland, draining glaciers 6, 10, 11, and 13. Kuannersuit Valley is composed of a primarily basaltic landscape, and numerous glacier streams here originate from the island’s largest ice cap, Sermersuaq, along with several valley and cirque glaciers (Žárský et al., 2018). Iceland was the second basaltic locality, and four sites were sampled: Sólheimajökull (outlet to Mýrdalsjökull), Skaftafellsjökull (south outlet to Vatnajökull), Eyjabakkajökull (north outlet to Vatnajökull), and Kaldalónsjökull (outlet to Drangajökull) (Björnsson et al., 2000; Tweed et al., 2005). Next, six localities were sampled on Svalbard. Two glaciers, Nansenbreen and Sefströmbreen, are located in Isfjorden, while Ebbabreen is located in Petuniabukta, and Midtre Lovénbreen near Ny-Ålesund, Kongsfjorden (Hagen et al., 1993). Lastly, cold-based glaciers Longyearbreen and Foxfonna were sampled near Longyearbyen. Three mainland Norway glaciers were sampled including Styggedalsbreen, Bøverbreen, and Austerdalsbreen, all of which are situated upon gneiss bedrock (Mateos-Rivera et al., 2016). Styggedalsbreen and Bøverbreen are located in the alpine Jotunheimen region, while Austerdalsbreen is an outlet glacier of Jostedalsbreen Ice Cap (Andreassen et al., 2012). Four outlet glaciers of the Juneau Icefield were sampled in coastal southeast Alaska; Herbert, Eagle, Lemon, and Mendenhall (Hood and Berner, 2009). Both Lemon Creek and Eagle River were sampled several km downstream due to inaccessibility, while Herbert and Mendenhall meltwater was sampled at the glacier snout. All four glaciers are underlain by felsic igneous intrusive bedrock. Finally, three outlet glaciers of the Greenland Ice Sheet (GrIS) were sampled, all of which drain Precambrian shield bedrock composed of Archaean gneiss and granite (Henriksen et al., 2009). Leverett Glacier was sampled several meters from its portal (Kohler et al., 2017), while Russell Glacier was sampled several hundred meters from its last glacial contact, upstream of the confluence with the Leverett River. Lastly, Qinnguata Kuussua, which drains the large Ørkendalen and Isorlersuup glaciers south of Leverett Glacier, was sampled immediately upstream of its confluence with the Akuliarusiarsuup Kuua to form the ‘Watson River’ near the town of Kangerlussuaq (Cameron et al., 2017).

**TABLE 1:** Physical characteristics of meltwater streams.

**Glacier**	**Region**	**Date sampled**	**Glacier area (km^2^)**	**Latitude**	**Longitude**	**Elevation (m)**	**Vol. water filtered (mL)**	**Temperature (°C)**	**Conductivity (μS/cm)**	**pH**	**Total suspended solids (g/L)**
Herbert	Alaska	27-Jun-17	60^a^	58.539120°	−134.684540°	280	3 × 300	0.30	21.00	7.70	0.339
Mendenhall	Alaska	28-Jun-17	127^a^	58.438010°	−134.544570°	113	3 × 300	0.20	18.00	8.61	0.219
Lemon	Alaska	29-Jun-17	15^a^	58.364320°	−134.478740°	20	3 × 360	5.30	38.00	7.81	0.025
Eagle	Alaska	29-Jun-17	53^a^	58.528640°	−134.805680°	6	3 × 480	3.70	18.00	8.07	0.050
Leverett	GrIS	5-Sep-17	1200^b^	67.064770°	−50.162940°	314	3 × 300	0.00	30.00	9.32	0.963*
Russell	GrIS	6-Sep-17	120^c^	67.076780°	−50.276820°	182	3 × 300	0.00	35.00	8.85	0.5*
Qinnguata Kuussua	GrIS	7-Sep-17	1800^d^	67.016170°	−50.653090°	42	3 × 300	0.00	33.00	8.75	NA
Sólheimajökull	Iceland	16-Aug-16	47^e^	63.534833°	−19.352194°	275	3 × 300	NA	48.00	8.78	0.500
Skaftafellsjökull	Iceland	17-Aug-16	100^f^	64.028667°	−16.932667°	153	3 × 250	NA	30.50	9.38	0.253
Eyjabakkajökull	Iceland	18-Aug-16	110^g^	64.666250°	−15.723694°	939	3 × 300	NA	5.60	8.24	0.283
Kaldalónsjökull	Iceland	20-Aug-16	37^g^	66.117611°	−22.287750°	318	3 × 500	NA	6.50	8.50	0.284
Styggedalsbreen	Norway	22-Sep-16	2.02^h^	61.488306°	7.880444°	1307	3 × 300	0.70	3.00	7.5**	0.009
Austerdalsbreen	Norway	24-Sep-16	19.85^h^	61.588500°	6.995333°	490	3 × 300	0.30	25.00	6.7**	0.026
Bøverbreen	Norway	25-Sep-16	6.75^h^	61.556694°	8.049500°	1432	3 × 300	0.70	2.00	6.1**	0.207
Glacier 6	Qeqertarsuaq	4-Aug-15	1.5^i^	69.715833°	−53.441617°	907	3 × 600	0.10	7.90	7.20	0.107
Glacier 10	Qeqertarsuaq	6-Aug-15	7^i^	69.766717°	−53.413400°	765	3 × 300	0.10	7.70	8.70	0.827
Glacier 11	Qeqertarsuaq	6-Aug-15	9.7^i^	69.784050°	−53.427200°	782	3 × 300	2.20	9.90	6.90	0.194
Glacier 13	Qeqertarsuaq	9-Aug-15	18^i^	69.801817°	−53.375900°	798	3 × 300	0.10	9.00	7.52	0.156
Midtre Lovénbreen***	Svalbard	19-Jul-16	6^j^	78.895567°	12.069350°	50	3 × 600	0.9	38.97	8.85	0.746
Nansenbreen	Svalbard	2-Aug-16	45.1^j^	78.353145°	14.075243°	154	2 × 150, 1 × 200	0.90	70.00	7.21	2.354
Sefströmbreen	Svalbard	4-Aug-16	155^j^	78.719740°	14.374894°	158	3 × 200	0.80	108.00	8.25	0.902
Ebbabreen	Svalbard	6-Aug-16	25^j^	78.726805°	16.794599°	288	3 × 300	0.60	112.00	7.60	0.363
Longyearbreen	Svalbard	10-Aug-16	4^j^	78.189720°	15.533134°	340	3 × 200	NA	210.00	8.37	0.599
Foxfonna	Svalbard	5-Aug-17	3.95^j^	78.15243°	16.10879°	425	1 × 100	0.10	25.00	8.10	NA

Estimates of glacier area is given with relevant citations: ^*a*^estimated from Hood and Berner (2009), ^*b*^Palmer et al. (2011), ^*c*^Van de Wal and Russell (1994), ^*d*^Lindbäck et al. (2015), ^*e*^Björnsson et al. (2000), ^*f*^Tweed et al. (2005), ^*g*^Björnsson et al. (2003), ^*h*^Andreassen et al. (2012), ^*i*^Žárský et al. (2018), ^*j*^Hagen et al. (1993). *Averaged from samples taken over 2018 summer. **Measurements from August 2018. ***Data is averaged from 3 days around the sampling day (i.e., 16, 18, and 21 July 2016).

### Sampling

At each stream, three replicate microbiological samples were taken from the thalweg of the water column using a sterile syringe (except Foxfonna, where only one replicate was taken). Water was passed through Sterivex filters (0.22 μm; Millipore, Billerica, MA, United States) until they clogged, which was between 50 and 600 mL, with most having at least 300 mL (Table 1). Filters were flushed of water, filled with nucleic acid preservation buffer (LifeGuard, MO BIO, Carlsbad, CA, United States), and promptly frozen at −20°C. Given that time of day may have a strong influence on the hydrology of glacial systems (longer residence-time water may be disproportionately released at low flows), stream sampling was undertaken to roughly correspond with diurnal peaks in runoff if possible.

### Chemical Analyses

Physical and hydrochemical characterization was conducted concurrently with microbial cell collection as described previously (Žárský et al., 2018). Briefly, conductivity and pH were measured *in situ* at each stream with either a Multi 3430 digital pH and conductivity meter (WTW, Weilheim, Germany) or a low range Hanna Combo (Hanna Instruments, United States) pH/conductivity meter. Latitude/longitude and elevation were measured by handheld GPS, and estimates of glacier areas were derived from the literature (Table 1). Meltwater samples for nutrient and dissolved organic carbon (DOC) analyses were collected directly from the stream via sterile syringe. Nutrient samples were filtered through 0.45 μm polyethersulfone GD/XP syringe filters (Whatman) into acid-washed 30 ml Nalgene HDPE bottles and immediately frozen at −20°C. Nutrient concentrations were determined by using a LaChat QuikChem 8500 flow injection analyser for nitrate (NO_3_^–^; QuikChem Methods 10−107−04−1−B; LOD = 1 μg L^–1^ = 71 nM), ammonium (NH_4_^+^; 10−107−06−1−Q; LOD = 8 μg L^–1^ = 571 nM) and soluble reactive phosphorus (SRP; 31-115-01-1-I; LOD = 1 μg L^–1^ = 32 nM). DOC samples were filtered through Whatman Puradisc AQUA syringe filters (cellulose acetate, 0.45 μm) into acid-washed 30 ml Nalgene HDPE bottles and frozen. DOC concentrations were determined using a Shimadzu TOC-L analyzer (Shimadzu, Kyoto, Japan) with high sensitivity catalyst (LOD for DOC = 20 μg L^–1^ = 1.7 μM).

Dissolved major ions (F^+^, Na^+^, K^+^, Mg^2+^, Ca^2+^, Cl^–^, SO_4_^2–^, and HCO_3_^–^) and dissolved silica (DSi) were sampled by taking meltwater from the thalweg with a clean, 1 l Nalgene bottle triple-rinsed with stream water. The water was filtered within 24 h through a 47 mm 0.45 μm cellulose nitrate filter membrane (Whatman) mounted on a clean Nalgene filter tower. Samples were stored in 30 ml HDPE Nalgene bottles, and kept refrigerated (∼4°C). Major ions were analyzed by ion chromatography on a Thermo Scientific Dionex ICS5000 + capillary system as described by Hawkings et al. (2015), with HCO_3_^–^ estimated from charge deficit (Tranter et al., 2002), and DSi measured using a LaChat QuikChem 8500 flow injection analyzer (QuikChem Method 31-114-27-1-D) as described by Hawkings et al. (2017). Pre-weighed filters were used to determine total suspended solids (TSS) after drying the filters in an oven at 50°C overnight, re-weighing, subtracting the filter weight and normalizing by the water volume that passed through (measured using a measuring cylinder and usually ∼300–500 mL). See Žárský et al. (2018) for further notes on analytical precision and accuracy.

### Chemical Indices

From our geochemical data, we calculated two indices that have been used previously in interpreting patterns in weathering and hydrology (e.g., Dubnick et al., 2017b). The sulfate mass fraction (SMF) is defined as the concentration of sulfate (SO_4_^2–^), divided by the sum of sulfate and bicarbonate (HCO_3_−; Brown et al., 1996; Tranter et al., 2002). High SMF (e.g., >0.5) values indicate that a larger proportion of protons are coming from sulfide oxidation compared to carbonation reactions, and is thus an indication of the influence of carbonation versus sulfide oxidation as proton/HCO_3_^–^ sources (Wadham et al., 2004). Complementing this, we also calculated a divalent/monovalent (DiMo) ratio of major cations: Ca^2+^ + Mg^2+^/Na^+^ + K^+^. This ratio is a crude approximation of the degree of carbonate dissolution versus silicate dissolution (Wadham et al., 2010). Higher DiMo values are likely to be more associated with carbonate weathering and a channelized drainage system, whereas lower numbers may be proportionally high in contributions from the distributed drainage system (Wadham et al., 2010; Dubnick et al., 2017b). All values were converted to μeq L^–1^ before calculation.

### Nucleic Acids Extraction, Quantification, and Sequencing

DNA from the suspended sediment samples was extracted, amplified, and sequenced identically as in Žárský et al. (2018). Briefly, DNA was extracted using the PowerWater Sterivex DNA Isolation Kit (MO BIO) following the manufacturer’s protocol. Extracted DNA was quantified using a Qubit fluorometer and Qubit dsDNA HS Assay Kit (Invitrogen, Carlsbad, CA, United States). Template DNA samples were shipped to the Mr. DNA laboratory (Shallowater, TX, United States) where 16S rRNA gene V4 region primers 515f/806r (Caporaso et al., 2011) with barcode on the forward primer were used in a 28 cycle PCR using the HotStarTaq Plus Master Mix Kit (Qiagen, Hilden, Germany) with an initial melt step of 94°C for 3 min, followed by 28 cycles of 94°C for 30 s, 53°C for 40 s, and 72°C for 1 min. After amplification, PCR products were checked in 2% agarose gel and the samples were pooled in equimolar proportions. Pooled samples were purified using calibrated Ampure XP beads. Sequencing was performed on an Illumina MiSeq platform following the manufacturer’s guidelines. The quality checked dataset is available in the MG-RAST database (Meyer et al., 2008) under the accession number MGP92375, and representative sequences of selected OTUs were given accession numbers MN880326-MN880375 in GenBank.

### Bioinformatic Analysis

Sequence data were analyzed by the pipeline SEED v2.0.4 (Větrovský et al., 2018). Paired ends were joined by fastq-join (Aronesty, 2011), and all sequences with mismatches in tags were removed from the dataset. Chimeras were detected, and the non-chimeric sequences were clustered into operational taxonomic units (OTUs) using UPARSE implemented in USEARCH 8.1.1861 (Edgar, 2013), with a 97% similarity threshold. The consensus from each OTU was constructed from a MAFFT alignment (Katoh and Standley, 2013), based on the most abundant nucleotide at each position. Singletons, chloroplasts, and mitochondria were removed, and OTUs identified as obvious PCR contaminants (i.e., human pathogens and symbionts, organisms strikingly incompatible with the glacier environment, and known contaminants of DNA isolation kits) were deleted. The resulting reads ranged from 22,336 to 113,601 per sample (mean = 64,233), and the dataset was rarefied to the lowest number (22,336). The 50 most abundant OTUs were identified against the SILVA nr. 132 database in Mothur (Schloss et al., 2009), and their putative metabolisms and ecological roles were assessed by megaBLAST and BLASTn algorithms against the GenBank nt/nr database. The characteristics of described species were accepted for OTUs showing sequence similarity >97% with these species. Finally, to calculate un-weighted and weighted UniFrac distances, a phylogenetic tree was created with RAxML (Stamatakis, 2014) and included the top 1,371 OTUs by abundance. The resulting new dataset was rarefied to the minimum number of reads (21,518) and was used in all ordination analyses.

### Statistical Analyses

To visualize differences in environmental variables between regions, we performed principle components analysis (PCA) with physical and hydrological variables hypothesized to have microbiological relevance using the *ggbiplot* package (Vu, 2011) in R. Variable distributions were investigated by plotting histograms, and were log_10_-transformed if necessary to create a normal distribution.

In order to ascertain differences in assemblage structure between regions, diversity indices (#OTUs, Chao1, and Shannon) were calculated for each sample using the full rarefied dataset and compared using Tukey’s Honest Significant Differences test (TukeyHSD). We then created unconstrained ordinations (principle coordinates analysis; PCoA) to evaluate variability between samples and sites using both un-weighted (presence/absence based) and weighted (abundance based) UniFrac distances on the unfiltered, untransformed subsampled dataset. The significance of geographical region on assemblage structure was tested by using a permutational multivariate analysis of variance (PERMANOVA) using the *adonis()* function in the *vegan* package (Oksanen et al., 2018). This was followed by a homogeneity of dispersion test (i.e., to see if regional groupings have statistically similar/dissimilar dispersions) conducted with the *betadisper()* function in *vegan*. Lastly, to visualize differences in the distribution of particularly influential OTUs, the top 50 OTUs by abundance were plotted (averaged by site and log_10_ + 1-transformed) in a heatmap. A dendrogram was produced with the *heatmap.2()* function in the *gplots* package (Warnes et al., 2019) using the ‘average’ clustering method and Euclidean distance. Significant clusters were identified using the *simprof()* function in the *clustsig* package (Whitaker and Christman, 2014), with identical clustering and distance methods described above, and with transformation = “identity” and alpha = 0.000001.

Distance-based redundancy analysis (dbRDA) models were then created for both the weighted and un-weighted UniFrac datasets to find the most parsimonious combination of environmental variables to explain variability in assemblage structure across all sites. Quinnguata Kuusua was assigned the same TSS value as for Leverett River given the similarity of the catchment size and close geographical proximity. Other sites/samples where environmental data were missing and could not be confidently substituted from other sources were removed from analysis (i.e., Foxfonna and Midtre Lovénbreen; Table 2). Instances where solute concentrations where below detection (e.g., DOC, Table 2) were replaced with half the detection limit value. Candidate models were constructed by including only environmental variables with variance inflation factors less than or equal to 5 to avoid including redundant, collinear parameters (SMF and DiMo were positively correlated with SO_4_^2–^ and negatively correlated with SRP, and because of the presumed greater biological relevance of the latter variables, the former were excluded from analyses). These included log_10_-transformed glacier elevation, area, latitude, pH, DSi, DIN, Cl^–^, DOC, TSS, SRP, and SO_4_^2–^. The best combination of variables for each of the un-weighted and weighted datasets was then isolated through backward selection using the *ordistep()* function in *vegan*. Significance of the full model, as well as individual terms, was assessed using the *anova()* function. In order to assess relationships between environmental variables and individual OTUs, Pearson correlation coefficients were calculated between the same environmental variables included in dbRDA candidate models and the top 50 OTUs using the *cor()* function in R. Heatmaps and dendrograms were subsequently generated using the *heatmap.2()* function, and significant clusters calculated as described above.

**TABLE 2:** Hydrochemical characteristics of meltwater, including nutrient concentrations, major cations and anions, dissolved organic carbon (DOC), sulfate mass fraction (SMF), and the divalent: monovalent ratio (DiMo).

**Site**	**SRP**	**NH_4_^+^**	**NO_2_^–^ + NO_3_^–^**	**DIN**	**DSi**	**F^–^**	**Cl^–^**	**SO_4_^2–^**	**Na^+^**	**K^+^**	**Mg^2+^**	**Ca^2+^**	**HCO_3_^–^**	**DOC**	**SMF**	**DiMo**
Herbert	2.5	0.0	12.1	12.1	378.4	80.4	234.1	2025.0	345.9	580.4	234.7	2138.3	6110.4	198.4	0.30	4.22
Mendenhall	2.4	3.0	14.4	17.4	288.2	77.5	283.3	1949.7	333.1	553.9	226.5	1911.2	5332.0	108.1	0.32	3.98
Lemon	1.3	0.0	33.8	33.8	690.7	85.1	272.8	2775.8	403.4	627.9	369.2	4481.6	13075.9	204.8	0.21	7.56
Eagle	0.6	0.0	16.1	16.1	516.7	75.5	370.3	667.7	463.7	493.6	249.3	1780.3	6807.8	227.9	0.11	3.33
Leverett	5.6	0.0	15.5	15.5	870.2	93.8	155.1	3333.4	1259.7	1008.9	357.3	2519.7	9424.1	218.1	0.31	1.92
Russell	2.3	34.1	23.7	57.8	986.7	85.7	148.5	3184.4	786.8	835.3	553.3	3379.1	11670.4	412.5	0.26	3.85
Qinnguata Kuussua	5.3	5.8	23.7	29.5	971.2	89.6	188.6	3308.3	1124.6	1007.1	462.9	2902.9	10739.3	233.0	0.28	2.45
Sólheimajökull	40.8	4.9	13.3	18.2	2661.7	540.3	2007.0	1928.3	5461.9	449.3	721.9	3217.0	20975.5	62.9	0.10	0.88
Skaftafellsjökull	25.7	8.9	27.1	36.0	1104.6	445.3	1785.3	739.2	3203.1	109.6	155.0	2166.3	10603.5	56.8	0.08	0.85
Eyjabakkajökull	8.8	5.0	5.7	10.7	464.5	420.0	94.5	140.0	400.8	29.2	101.3	529.6	1541.0	<LOD	0.10	1.91
Kaldalónsjökull	9.3	6.3	6.2	12.5	381.1	228.9	535.2	469.2	916.8	46.3	118.3	484.0	2320.5	<LOD	0.20	0.83
Styggedalsbreen	3.8	8.6	31.6	40.2	300.7	16.2	62.1	272.5	132.0	100.1	153.8	370.8	1902.6	105.4	0.15	3.75
Austerdalsbreen	2.2	5.5	56.4	61.9	747.3	174.4	234.0	6702.7	442.9	527.4	154.5	3212.0	3070.4	111.5	0.73	5.28
Bøverbreen	6.4	6.8	<LOD	6.8	139.1	21.1	77.7	433.0	119.2	248.1	54.3	167.7	735.0	84.0	0.43	1.11
Glacier 6	9.1	3.5	0.0	3.5	441.9	6.7	390.9	179.7	564.8	42.8	85.2	710.6	3233.7	192.3	0.07	1.66
Glacier 10	17.5	18.2	0.0	18.2	653.1	4.4	327.1	226.1	703.8	42.7	1335.3	751.5	10060.0	151.8	0.03	4.65
Glacier 11	17.7	25.9	0.0	25.9	221.7	5.2	157.9	102.1	253.9	41.2	91.1	185.2	1341.0	134.8	0.09	1.38
Glacier 13	29.8	9.5	29.5	39.0	974.1	5.9	226.3	206.0	1250.7	42.7	44.2	522.7	4498.9	155.5	0.05	0.54
Midtre Lovénbreen*	NA	NA	24.5	NA	NA	NA	205.0	6362.7	703.3	350.0	1068.0	6923.7	20421.3	269.6	0.72	11.01
Nansenbreen	2.1	13.0	4.2	17.2	128.1	143.8	83.4	14632.4	352.8	391.7	2027.8	13000.9	32105.9	169.7	0.37	32.16
Sefströmbreen	0.3	6.2	4.6	10.8	92.9	288.3	57.6	18922.1	108.8	104.9	998.1	19717.4	40419.1	84.4	0.37	143.75
Ebbabreen	0.0	12.5	15.2	27.6	85.2	50.8	1220.1	28347.7	1143.0	412.7	1576.7	19307.5	32080.8	80.9	0.53	18.14
Longyearbreen	1.2	31.7	309.6	341.3	372.2	53.9	1579.2	86419.4	15841.2	688.7	10156.4	13895.7	23665.5	187.7	0.82	2.16
Foxfonna	2.0	15.0	28.7	43.7	194.2	84.3	456.4	6119.5	1992.8	580.4	375.2	142.1	5733.1	640.2	0.58	0.37

All concentrations are reported in parts per billion (ppb), and SMF and DiMo ratios were converted to μeq L^−1^ before calculation. *Data is averaged from 3 days around the sampling day (i.e., 16, 18, and 21 July 2016).

Unless otherwise stated, significance was designated at α = 0.05, adjusted (Adj. *R*^2^ values are reported, and all statistics and figures were generated using the R statistical environment (R Core Team, 2017), primarily using functions available within the phyloseq package (McMurdie and Holmes, 2013).

## Results

### Differences in Glacier and Meltwater Characteristics

**FIGURE 2:**
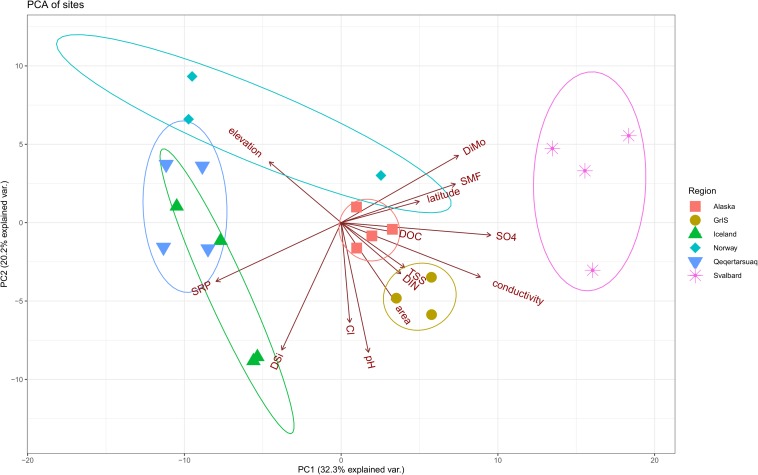
Principle components analysis (PCA) showing differences in selected environmental variables by region. Alaska sites are shown in red squares, the Greenland Ice Sheet (GrIS) in gold circles, Iceland in green triangles, Norway in turquoise diamonds, Qeqertarsuaq in blue upside-down triangles, and Svalbard in purple asterisks. Sites with missing environmental data are excluded (i.e., Foxfonna and Midtre Lovénbreen, see Tables 1, 2). Ellipse probabilities are set to 0.8.

Regional differences were observed in the measured physical and chemical characteristics of glacial meltwater (Figure 2, see Tables 1, 2 for a full summary). Glaciers from Norway and Qeqertarsuaq were sampled at the highest elevations, and samples from the GrIS had the largest catchment areas and TSS concentrations. Iceland and Qeqertarsuaq, both being basaltic localities, clustered together in the PCA, while other regions did not show substantial overlap (Figure 2). These streams had among the greatest SRP and DSi concentrations, and lowest DiMo ratios (indicating the predominance of silicate over carbonate weathering). Meltwater streams from Svalbard displayed comparatively high conductivities, as well as the greatest DiMo values and SO_4_^2–^ and DOC concentrations. SMF values were greatest in two of the Norway sites (Bøverbreen and Austerdalsbreen) and two of the Svalbard sites (Ebbabreen and Longyearbreen). Iceland, along with the GrIS outlet glaciers, also had the greatest pH values, while Alaskan and Norwegian glaciers had very low measured pH (6.1-7.5 for Norway; Table 1).

### Meltwater Assemblage Structure

Stream assemblages were dominated by the domain Bacteria, and all streams exported substantially less than 1% of Archaea by relative abundance. In total, 404 orders from 58 unique phyla were identified from the full, rarefied dataset. All samples were dominated by the phylum Proteobacteria, which had a mean relative abundance of 50.4%, and ranged from 13.3 to 67.2% (Supplementary Figure S1). Proteobacteria was followed in abundance by Bacteroidetes (mean = 16.2, range = 2.38–26.5%), and Actinobacteria (mean = 9.86%, range = 3.38–45.4%). Several sites also had notable proportions of Acidobacteria (mean = 3.06%, range = 0.10–9.58%) and Verrucomicrobia (mean = 4.86%, range = 0.049–20.9%), which were highest in abundance at Greenland sites (Qeqertarsuaq and GrIS). Cyanobacteria averaged 1.10% across all samples, and ranged from 0 to 10.2%. In terms of orders, Betaproteobacteriales (i.e., Betaproteobacteria) was the most common (mean = 33.6%, range = 4.97–50.6%), followed by Sphingobacteriales (mean = 4.93%, range = 0.31–11.3%), Chitinophagales (mean = 4.54%, range = 0.09–24.9%), and Micrococcales (mean = 4.01%, range 0.40–10.9%). Cytophagales (mean = 3.93%, range = 0.27–15.3%) and Verrucomicrobiales (mean = 3.18%, range = 0.02–20.4%) furthermore made up a substantial proportion of a few samples.

**FIGURE 3:**
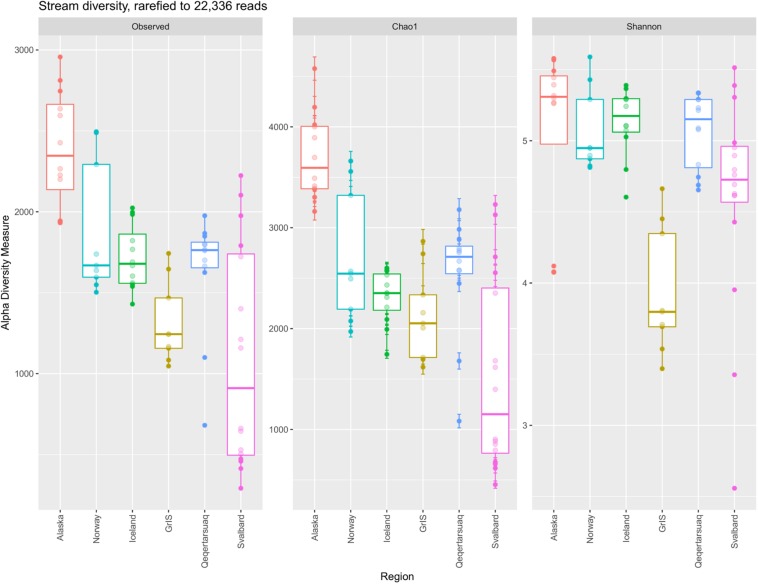
Boxplots comparing the observed number of OTUs, Chao1, and Shannon diversity between major (sub)Arctic regions. The dataset was rarefied to 22,336 reads, and regions (*x*-axis) are ordered by increasing latitude.

In total, 16,986 OTUs were observed in the full rarefied dataset. Of these, 150 were observed at all sites, and 1,313 were observed within all six regions. In contrast, 6,637 OTUs were present at one site only, and 8,056 were observed from one region only. Alaska had the most unique OTUs with 3,239 (∼32% of its total diversity), followed by Norway with 1,116, Iceland with 1,076, Qeqertarsuaq with 994, Svalbard with 934, and the GrIS with 697 (∼16% of its total diversity). Calculated alpha diversity metrics showed strong variability among regions (Figure 3), and differences were significant among all of Observed OTU richness (ANOVA, *F* = 13.63, *p* << 0.01), Chao1 (*F* = 17.33, *p* << 0.01), and Shannon diversity (*F* = 8.82, *p* << 0.01). Specifically, Alaskan streams had significantly greater Observed OTU richness and Chao1 values than all other regions (TukeyHSD, *p* < 0.01 for all comparisons), with the exception of Norway in the case of Observed OTUs (*p* = 0.11). Similarly, Iceland, Norway, and Qeqertarsuaq regions had significantly greater Observed OTU richness and Chao1 values in comparison to Svalbard (*p* < 0.03 for all comparisons). On the other hand, Shannon diversity was more similar between regions with the exception of the Greenland Ice Sheet, which had substantially lower values, and all regions had significantly greater values in comparison (*p* < 0.05 for all). When compared with latitude, Observed OTU richness (Adj. *R*^2^ = 0.42, *F* = 51.89, *p* << 0.01), Chao1 (Adj. *R*^2^ = 0.45, *F* = 57.38, *p* << 0.01), and Shannon diversity (Adj. *R*^2^ = 0.06, *F* = 5.06, *p* = 0.03) were all significantly and negatively correlated.

**FIGURE 4:**
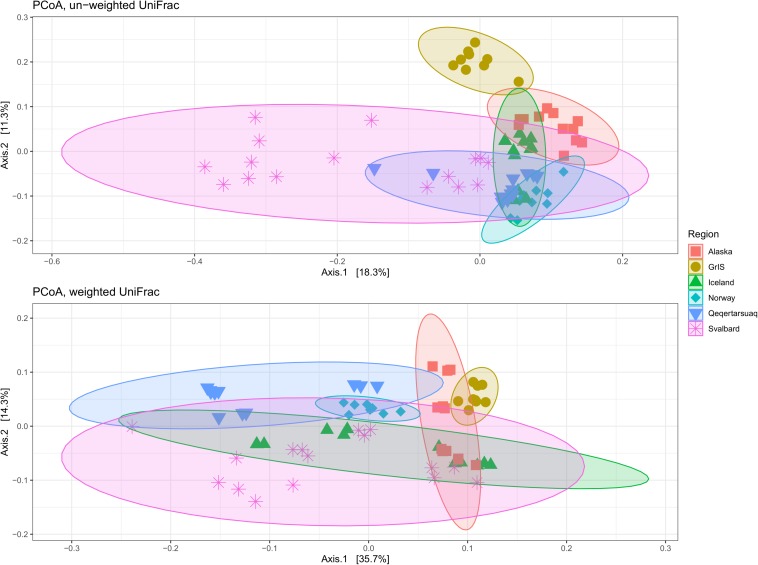
Principle coordinates analysis (PCoA) showing differences between geographic regions on un-weighted **(top)** and weighted **(bottom)** UniFrac distances. Alaska sites are shown in red squares, the Greenland Ice Sheet (GrIS) in gold circles, Iceland in green triangles, Norway in turquoise diamonds, Qeqertarsuaq in blue upside-down triangles, and Svalbard in purple asterisks. Colored circles indicate 95% confidence intervals of regional categories.

Principle coordinate analyses (PCoA) were conducted to assess relationships between assemblages across geographic regions. When un-weighted UniFrac distances were applied (i.e., OTUs receive equal weighting), 29.6% of the variability was explained by axis 1 and 2 combined (Figure 4). GrIS sites, and a subset of the Svalbard samples, clustered apart from other regions, while Norway, Iceland, Alaska, and Qeqertarsuaq samples formed an overlapping cluster. When tested with PERMANOVA, geographical regions were significant in explaining assemblage variability (*R*^2^ = 0.36, pseudo*F* = 7.18, *p* < 0.01), although dispersions were significantly different by region (pseudo*F* = 15.81, *p* < 0.01). When weighted UniFrac distances were used (i.e., accounting for abundance), axis 1 and 2 together explained 50.0% of the variability (Figure 4). All regions clustered closely together, with Qeqertarsuaq, GrIS, and a subset of Alaskan sites oriented more toward the top of the figure, and with a subset of Svalbard sites oriented toward the bottom. Application of the PERMANOVA test suggested that these regional groupings were also significant different (*R*^2^ = 0.47, pseudo*F* = 11.51, *p* < 0.01), although regions again significantly differed in their dispersions (pseudo*F* = 7.55, *p* < 0.01).

**FIGURE 5:**
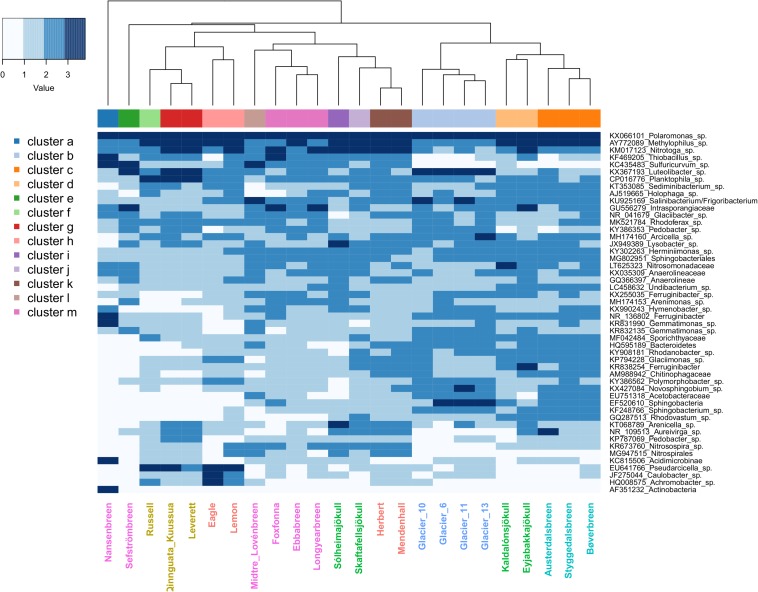
Heatmap showing the log_10_ + 1-transformed abundance of the top 50 OTUs averaged by glacier stream site for the full rarefied dataset. Higher values are indicated by greater shade intensity. Site labels are colored by region, with Alaska sites indicated by red, the Greenland Ice Sheet (GrIS) by gold, Iceland by green, Norway by turquoise, Qeqertarsuaq by blue, and Svalbard by purple. The column side bar indicates the 13 significant site clusters.

In order to gain insight into influential taxa driving patterns in the PCoA analyses, the top 50 OTUs by abundance were identified and averaged by site (see Supplementary Table S1 for full taxonomic and ecological information). Within sites, the top 50 OTUs collectively represented between 40 and 76% of the total number of reads in the full rarefied dataset (mean and median = 54%). When averages were plotted in a heatmap (Figure 5), multiple glaciers from the same region formed significant groups, but no glaciers from different regions significantly clustered together. In total, 13 significant clusters were formed, with Nansenbreen alone forming cluster a. Cluster b was formed by the Qeqertarsuaq sites (Glacier 6, 10, 11, and 13), and cluster c by the Norwegian sites (Austerdalsbreen, Bøverbreen, and Styggedalsbreen). Two of the Iceland sites, Kaldalónsjökull and Eyjabakkajökull, formed cluster d. Sefströmbreen and Russell glaciers both formed their own clusters, cluster e and f, respectively. Leverett and Qinnguata Kuusua from the GrIS formed cluster g, and Eagle and Lemon from Alaska formed cluster h. The remaining Iceland sites, Sólheimajökull and Skaftafellsjökull, clustered alone (clusters i and j, respectively). Alaskan glaciers Herbert and Mendenhall together formed cluster k, and Midtre Lovénbreen alone formed cluster l. Lastly, the remaining Svalbard sites, Foxfonna, Ebbabreen, and Longyearbreen, formed cluster m.

Three OTUs in particular were abundant at all sites, with the most common of these being *Polaromonas* sp. (Figure 5 and Supplementary Table S1). On average, *Polaromonas* sp. accounted for 15% of all reads, ranging from 3 to 28% across samples. This was followed by *Methylophilus* sp. with an average relative abundance of 6% (ranging 1–15%) and *Nitrotoga* sp. with 4% (ranging <1–23%). However, at lower abundances, regional microbial assemblages became more distinct. For example, Greenland sites (GrIS and Qeqertarsuaq) had higher abundances of the Verrucomicrobium *Luteolibacter* sp., and GrIS and the larger Alaskan rivers (Eagle Glacier and Lemon Glacier) had high abundances of *Pseudarcicella* sp., which was at low abundances at all other sites. Svalbard sites (as well as Mendenhall Glacier, Herbert Glacier, and a few others) had high abundances of sulfur oxidizers *Sulfuricurvum* sp. from the phylum Epsilonbacteraeota (i.e., Epsilonproteobacteria), and *Thiobacillus* sp. from the phylum Proteobacteria (Figure 5 and Supplementary Table S1). Finally, sites from the GrIS also had elevated abundances of *Planktophila* sp.

### Correlations With Environmental Variables

**FIGURE 6:**
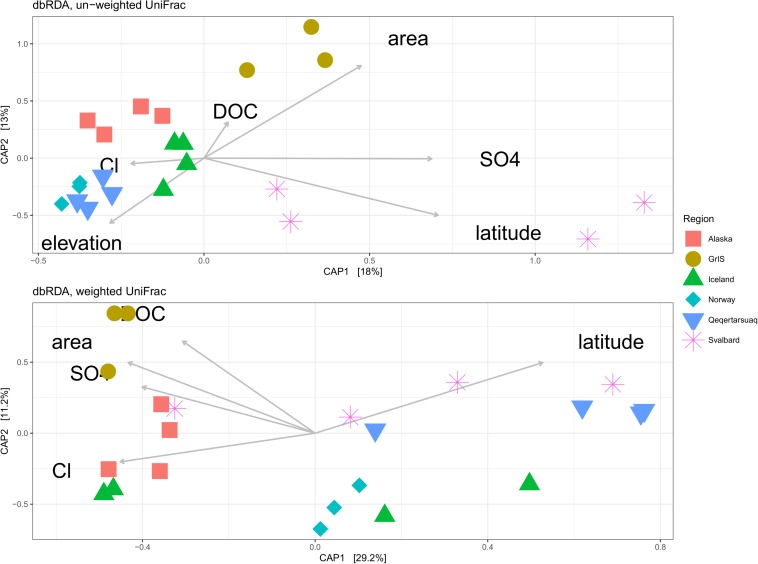
Distance-based redundancy analysis (dbRDA) showing the most parsimonious combination of explanatory variables for explaining assemblage structure for both un-weighted **(top)** and weighted **(bottom)** UniFrac datasets. Sites with missing metadata were excluded (i.e., Foxfonna and Midtre Lovénbreen, see Tables 1, 2). Alaska sites are shown in red squares, the Greenland Ice Sheet (GrIS) in gold circles, Iceland in green triangles, Norway in turquoise diamonds, Qeqertarsuaq in blue upside-down triangles, and Svalbard in purple asterisks.

We constructed dbRDA models to identify physical and chemical variables that best explain variability in exported microbial assemblage structure across sites (Figure 6). For the un-weighed UniFrac dataset, the most parsimonious model included elevation (*F* = 2.78, *p* = 0.01), Cl^–^ (*F* = 1.55, *p* = 0.06), DOC (*F* = 1.81, *p* = 0.03), SO_4_^2–^ (*F* = 3.84, *p* < 0.01), glacier area (*F* = 3.04, *p* < 0.01), and latitude (*F* = 3.48, *p* < 0.01). The *y*-axis explained 13.0% of the variability in the dataset, and was driven primarily by elevation and latitude toward the bottom, and glacier area toward the top, being most strongly associated with GrIS samples. The *x*-axis explained 18.0% of the variability, and was primarily driven by SO_4_^2–^ and latitude toward the right, corresponding mostly closely to Svalbard samples, and elevation and Cl^–^ concentrations toward the left, corresponding with Alaska, Norway, Iceland, and Qeqertarsuaq. The full model explained 31.0% of the variability, and was significant by ANOVA (*F* = 2.75, *p* < 0.01).

For the weighted UniFrac dataset (Figure 6), the most parsimonious model included DOC (*F* = 3.46, *p* < 0.01), glacier area (*F* = 3.73, *p* < 0.01), SO_4_^2–^ (*F* = 3.20, *p* = 0.01), Cl^–^ (*F* = 3.42, *p* < 0.01), and latitude (*F* = 6.20, *p* < 0.01). The *y*-axis explained 11.2% of the variability, and was predominantly driven by DOC and glacier area toward the top, being most closely associated with GrIS samples. The *x*-axis explained 29.2% of the variability, and was driven primarily by latitude toward the right and Cl^–^, SO_4_^2–^, and glacier area to the left. Svalbard and Qeqertarsuaq samples were most strongly oriented toward the right, while GrIS and Alaska were oriented toward the left. The full model explained 40.4% of the variability, and was significant by ANOVA (*F* = 4.00, *p* < 0.01).

**FIGURE 7:**
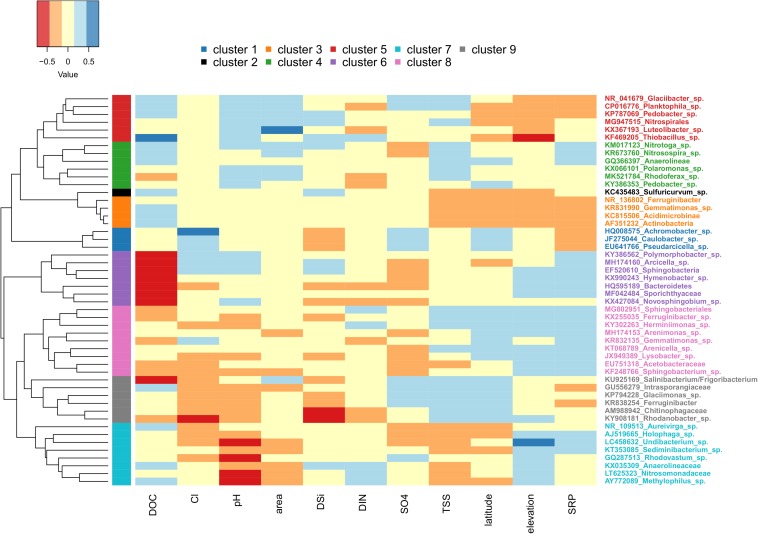
Heatmap showing the relationship between environmental variables (only those with variance inflation factors less than or equal to 5 included) and the top 50 OTUs. Sites with missing metadata were excluded (i.e., Foxfonna and Midtre Lovénbreen, see Tables 1, 2). Cool colors indicate high Pearson correlation coefficient values, and warm colors indicate low values. The row side bar indicates the nine significant OTU clusters.

The abundance of the top 50 OTUs was then compared with corresponding hydrochemical characteristics to determine possible drivers for common taxa (Figure 7). Based on these relationships, the row dendrogram split the top 50 OTU’s into nine significant clusters. Cluster 1 included *Achromobacter* sp., *Caulobacter* sp., and *Pseudarcicella* sp., which were positively correlated with latitude, Cl^–^, and SO_4_^2–^ and negatively correlated with SRP and DSi. Cluster 2 was formed by *Sulfuricurvum* sp. alone, and cluster 3 included four OTUs (*Ferrunginibacter*, *Gemmatimonas* sp., *Acidimicrobinae*, and *Actinobacteria*). Both clusters 2 and 3 were positively correlated with DOC, but negatively correlated with TSS, latitude, elevation, and SRP. Cluster 4 included several of the most common OTUs, such as *Polaromonas* sp., *Rhodoferax* sp., and *Nitrotoga* sp., and was (mostly) positively correlated with pH, glacier area, TSS, and SRP. Cluster 5 hosted some of the remaining abundant OTUs, such as *Luteolibacter* sp., *Thiobacillus* sp., and *Glaciibacter* sp., and were negatively related to latitude, elevation, and SRP, but positively correlated with DOC, pH, and glacier area. Cluster 6 was negatively correlated with DOC and SO_4_^2–^ concentrations, but positively correlated with SRP and elevation. Clusters 7 and 9, the former of which hosted the common *Methylophilus* sp., were both negatively correlated with pH and Cl^–^ overall. However, cluster 9 was positively correlated with TSS, SO_4_^2–^, and latitude, while cluster 7 showed the opposite relationships. Lastly, cluster 8 showed positive relationships with SRP, latitude, and elevation, but showed mixed relationships with the remaining variables (Figure 7).

## Discussion

As glacial melt rates continue to increase across the northern hemisphere (Zemp et al., 2019), a fuller understanding of the consequences of deglaciation is warranted. One of the most conspicuous of the anticipated effects will be the altered production of meltwater (e.g., Milner et al., 2017; Huss and Hock, 2018), along with associated changes in hydrologic pathways (e.g., meltwater generated further inland and at greater elevations, intensifying connectivity between supra- and subglacial habitats), which ultimately have the greatest relevance for determining the quantity and character of solute and particulate fluxes. Yet, while the physical and chemical changes accompanying deglaciation may be comparatively straightforward to predict, the biological consequences for glacial ecosystems are far less intuitive (Fell et al., 2017; Hotaling et al., 2017a), and generalizations are inherently difficult to make due to differences in glacier size, elevation, bedrock, thermal regime, vegetation, and precipitation patterns (e.g., Carnahan et al., 2019). By studying microbial assemblages exported by glacier meltwater streams, it may be possible to investigate microbial processes taking place in the overall glacial system, and assess changes in structure and export over time.

### Assemblage Structure

In this study, we performed a geographically broad survey of glacial streams from across the Arctic and sub-Arctic to investigate whether the composition of microbial assemblages is linked to differences in geographic location and/or the physical and chemical characteristics of meltwater. We found meltwater assemblages to have the same coarse structure reported from other glacier streams (taking into consideration updates to the Silva database), being dominated by the phyla Proteobacteria and Bacteroidetes (Sheik et al., 2015; Cameron et al., 2017; Dubnick et al., 2017a). Interestingly, we found that all glacier meltwater streams export a small subset of the same OTUs at high relative abundances. A species of *Polaromonas* was the most abundant OTU recovered from all sites in this study, and belongs to a genus exhibiting a ubiquitous, global distribution throughout the cryosphere (Darcy et al., 2011). While the ecological role of *Polaromonas* spp. has not been decisively resolved, they are thought to be generalists, able to utilize a wide variety of carbon substrates and survive inhospitable periods (possibly including long-range dispersal) through dormancy (Darcy et al., 2011; Franzetti et al., 2013). The other abundant OTUs included the methylotrophic *Methylophilus* sp. and nitrogen oxidizing *Nitrotoga* sp., both of which are genera commonly recovered from cold environments globally (Achberger et al., 2016; Goordial et al., 2016; Boddicker and Mosier, 2018).

Yet, assemblages were also regionally unique, with up to a third of all OTUs from a given region being exclusive to that region. Most of these unique OTUs were found within the rare biosphere (exhibiting less than ∼0.1% relative abundance; Lynch and Neufeld, 2015), which helps to explain differences observed between the un-weighed and weighted UniFrac analyses. Interestingly, the three regions with the greatest number of unique OTUs (Alaska, Norway, and Iceland) clustered tightly together in the un-weighted UniFrac ordination (which is more sensitive to differences in low-abundance OTUs) while regions with fewer unique OTUs (specifically Svalbard and GrIS) presented less overlap with other regions and a greater dispersion between sites. However, when weighted UniFrac distances were plotted (taking into consideration OTU abundances), there was much greater overlap among regions, reflecting the high relative abundances of the several aforementioned OTUs that were common to all sites. When a dendrogram was created to compare the relationships between sites and the top 50 OTUs, the ‘high elevation’ sites from Qeqertarsuaq, Iceland, and Norway were mostly oriented toward the right. These sites had relatively low relative abundances of sulfur oxidizers *Thiobacillus* and *Sulfuricurvum*, especially in comparison to the Svalbard sites, where SO_4_^2–^ concentrations are commonly high (Yde et al., 2008). Meanwhile, the larger rivers sampled from Alaska (Lemon Glacier and Eagle Glacier) and the GrIS clustered to the left, set apart by relatively high abundances of *Pseudarcicella* sp. and *Planktophila* sp. As both *Pseudarcicella* (e.g., Cruaud et al., 2019) and *Planktophila* (Lee and Eom, 2016) are relatively common freshwater genera, their elevated relative abundances are potentially an indicator of lateral freshwater inputs between the source glacier and sampled sites.

Furthermore, regions differed in their magnitude of exported diversity, and alpha diversity decreased with increasing latitude. This pattern is a well-known phenomenon for macro-organisms (i.e., the Latitudinal Diversity Gradient, e.g., Pianka, 1966; Rohde, 1992; Hillebrand, 2004), and has more recently been observed for microorganisms in other biomes, such as the ocean (Fuhrman et al., 2008; Raes et al., 2018). As argued for other systems, this pattern may be a function of geological age, greater productivity (either from more calendar days with solar radiation, or potentially greater fluxes of allochthonous organic carbon transported to glacier surfaces), or a higher mean air temperature, which could enhance supraglacial metabolic activity. However, the Latitudinal Diversity Gradient has seen mixed support in terrestrial soil bacterial communities (Fierer and Jackson, 2006; Chu et al., 2010), and more work will be necessary to validate this pattern and identify its drivers within glacial environments. Importantly, most of the taxa identified in this study are not endemic (Supplementary Table S1), but bacteria with (putatively) cosmopolitan distributions. Thus, differences in among-site diversity are likely more attributable to the diversity of available niches rather than geographical isolation or dispersal limitation.

### Relationships With Meltwater Characteristics

In addition to possible spatial patterns, we also hypothesized that assemblage structure would be related to the physical and chemical characteristics of the meltwater, reflecting dominant hydrologic processes as well as potential energy sources. We found that the most parsimonious models for both the un-weighted and weighted dbRDA analyses included DOC, SO_4_^2–^, latitude, glacier area, and Cl^–^, indicating that similar factors are responsible for determining both the taxa present as well as their relative abundance in glacier meltwater. However, the magnitude of their importance differed between un-weighted and weighted analyses, and may therefore represent different mechanisms of influence. Specifically, physical variables such as latitude, glacier area, SO_4_^2–^, and elevation showed a high level of influence in the un-weighted analysis. These variables potentially represent a gradient of physical habitat types, which may in turn correspond to niche and taxonomic diversity. In contrast, Cl^–^, SO_4_^2–^, and DOC exhibited a higher degree of influence in the weighted dbRDA analysis, and may reflect differences in the availability of necessary solutes/resources, which may allow a subset of taxa to proliferate.

Comparisons between the identity and inferred metabolisms of the top 50 OTUs (Supplementary Table S1) with environmental variables were made to help further disentangle factors structuring assemblages between sites and regions. Furthermore, we reasoned that clusters showing higher correlations with proxies generally associated with greater rates of subglacial weathering (e.g., TSS, SRP, pH, and DSi) may indicate a proportionately greater subglacial source of cells. A subset of the clusters indeed seemed reasonable from this perspective. For example, the OTUs composing clusters 4 and 5 were identified as aerobic autotrophs, and showed an overall positive correlation with pH, glacier area, and TSS, suggesting that these OTUs are disproportionately sourced from subglacial habitats (potentially utilizing basal melt as an oxygen source). Adding to this interpretation, *Thiobacillus* from cluster 5 is a commonly observed subglacial genus (e.g., Achberger et al., 2016), and cluster 4 included *Rhodoferax* sp., which was previously found to dominate subglacial sediment samples from Qeqertarsuaq (Žárský et al., 2018). However, other clusters presented more ambiguous associations. For example, clusters 2 and 3 were disproportionately composed of OTUs inferred to be anaerobic, and while their positive relationship with DSi may collectively suggest a greater subglacial source, their negative relationships with TSS and SRP make this argument less strong. Similarly, members of clusters 6 and 8 are almost all inferred to be aerobic heterotrophs, yet showed mixed relationships with all of TSS, DSi, and pH. Thus, in the majority of cases, the origin of exported OTUs from the glacier environment was not possible to resolve with our cluster analysis.

Correlations between individual OTUs and the meltwater chemistry were also sometimes counterintuitive. For example, autotrophic sulfur oxidizers *Thiobacillus* sp. and *Sulfuricurvum* sp. correlated positively with DOC, but showed no relationship with SO_4_^2–^. Similarly, inferred nitrifiers (*Nitrotoga* sp. and *Nitrosospira* sp.) and nitrogen reducers (*Glaciimonas* sp., and *Intrasporangiaceae*) showed no relationship with DIN. Clusters 6 and 8, which were predominately composed of aerobic heterotrophs, were strongly negatively correlated with DOC (though they were positively correlated with SRP). A final example is from the Nansenbreen glacier, where sulfur oxidizers represent up to 25% of the top 50 OTUs, yet sulfate is only slightly elevated compared with other sites. While it is possible that comparisons with different chemical species might yield different results (e.g., H_2_S instead of SO_4_^2–^), and we understand caution should be exercised in making conclusions from the inferred metabolisms of clustered OTUs, we still expected more robust relationships with some of these more specific taxa with solutes corresponding to their metabolisms.

One possible explanation for these results may be due to collinearity within sites. Sites strongly clustered within regions in the PCA analyses, suggesting strong site-related variability in environmental variables. Furthermore, it is likely that some samples play a disproportionate role in driving OTU responses given insufficient gradients for some variables. For example, Svalbard had the greatest values for many variables (including latitude, chemical indices, and conductivity), which set this region apart in the PCA, and thus generally represented the higher range of abiotic characteristics in individual comparisons. Therefore, it is difficult to say if correlations in general are an indicator of hydrologic processes, reflect biogeographic/regional patterns, or are entirely spurious. Furthermore, glaciers were sampled at different stages of hydrological development. As discussed previously by Hatton et al. (2019b), our ‘spot sampling’ approach may make potential signals difficult to identify and/or interpret due to their being taken out of the hydrological context of the site. Future efforts might find very different patterns and relationships with hydrochemical variables if samples are taken throughout different points in the year (Sheik et al., 2015; Dubnick et al., 2017a) or over a greater selection of sites.

Another confounding factor is the problem of scale and chemical mixing. In general, while glaciers host broad supra- and subglacial habitats, there are also ‘microhabitats’ within these heterogeneous domains with their own specific energy sources and chemical signatures, which is highlighted by our observation of a few taxa at relatively high abundances in a subset of sites. However, as solutes and particulates are collected by meltwater, they are diluted, and thus the unique physico-chemical characteristics of microhabitats (as well as supra- and subglacial chemical signals) can be ‘averaged away’, making them undetectable through bulk meltwater analyses. However, these microhabitats may be important hotspots of subglacial life given that any energy source is likely to have a large impact in an otherwise energy-limited environment. We argue that one considerable strength of analyzing the microbial assemblages of meltwater is that it may be possible to detect these microhabitats with the rationale that any spot with high microbial productivity is likely to have an elevated signature in the mixed community structure, though it may not necessarily be reflected by meltwater characteristics. Thus, OTUs that cannot be explained by bulk meltwater chemistry may actually be indicators of these otherwise undetectable microhabitats.

## Conclusion

Our results suggest that glaciers export both shared cosmopolitan taxa that dominate assemblages, as well as microbes unique to particular regions and sites, and highlight the heterogeneous nature of glacial environments their associated microbiota. Greater exported diversity was uncovered at lower latitudes, which are also the most prone to physical reduction from climate change, and are thus the most likely to experience broad changes in the diversity of microbial export in the future. Furthermore, we found some assemblages contain individual OTUs with distinct metabolic signals, likely reflecting spatially confined energy sources that have small effects on overall water chemistry, but a great influence on meltwater assemblage structure. Thus, rather than reflecting biogeochemical characteristics of meltwater, we found microbial cells instead provide important information about glacier habitats that are essentially impossible to resolve by analyzing bulk meltwater chemistry alone. Given the contributions of glacier exports to stream microbial diversity, it follows that post deglaciation, a substantial source of this diversity may disappear, although the viability and potential functional roles performed by exported microbes (e.g., competition, nutrient cycling, genes for exchange, etc.) are poorly explored. Nonetheless, this work suggests that exported microbial cells show promise as biological tracers for investigating hydrological processes, exploring the heterogeneous nature of subglacial habitats, and monitoring changes in glaciated watersheds.

## Data Availability Statement

Sequence data are available through the MG-RAST database under the accession number MGP92375, and representative sequences of selected OTUs were given accession numbers MN880326-MN880375 in GenBank. All other data are available upon request to the corresponding author.

## Author Contributions

TK, JŽ, JY, and MS conceived of the project. TK, PV, JŽ, JY, LF, GL-G, EH, KC, and MS performed the fieldwork and collected samples. TK, PV, GL-G, JŽ, and LF performed molecular laboratory work. LF performed bioinformatics analyses. JRH and JEH performed all geochemical analyses. TK analyzed the data and wrote the manuscript with significant input and editing from all co-authors.

## Conflict of Interest

The authors declare that the research was conducted in the absence of any commercial or financial relationships that could be construed as a potential conflict of interest.
