# Supplementary material for: Patterns in Microbial Assemblages Exported From the Meltwater of Arctic and Sub-Arctic Glaciers
Source: Front Microbiol. 2020 Apr 15;11:669. doi: 10.3389/fmicb.2020.00669 (PMC7174618; doi:10.3389/fmicb.2020.00669)
Supplement: Supplementary file 1 [file Data_Sheet_1.DOCX]

Supplementary Material

**Supplementary Figure 1.** Boxplots showing the average relative abundances of the top 25 phyla (top) and orders (bottom) compiled from all sites and samples.


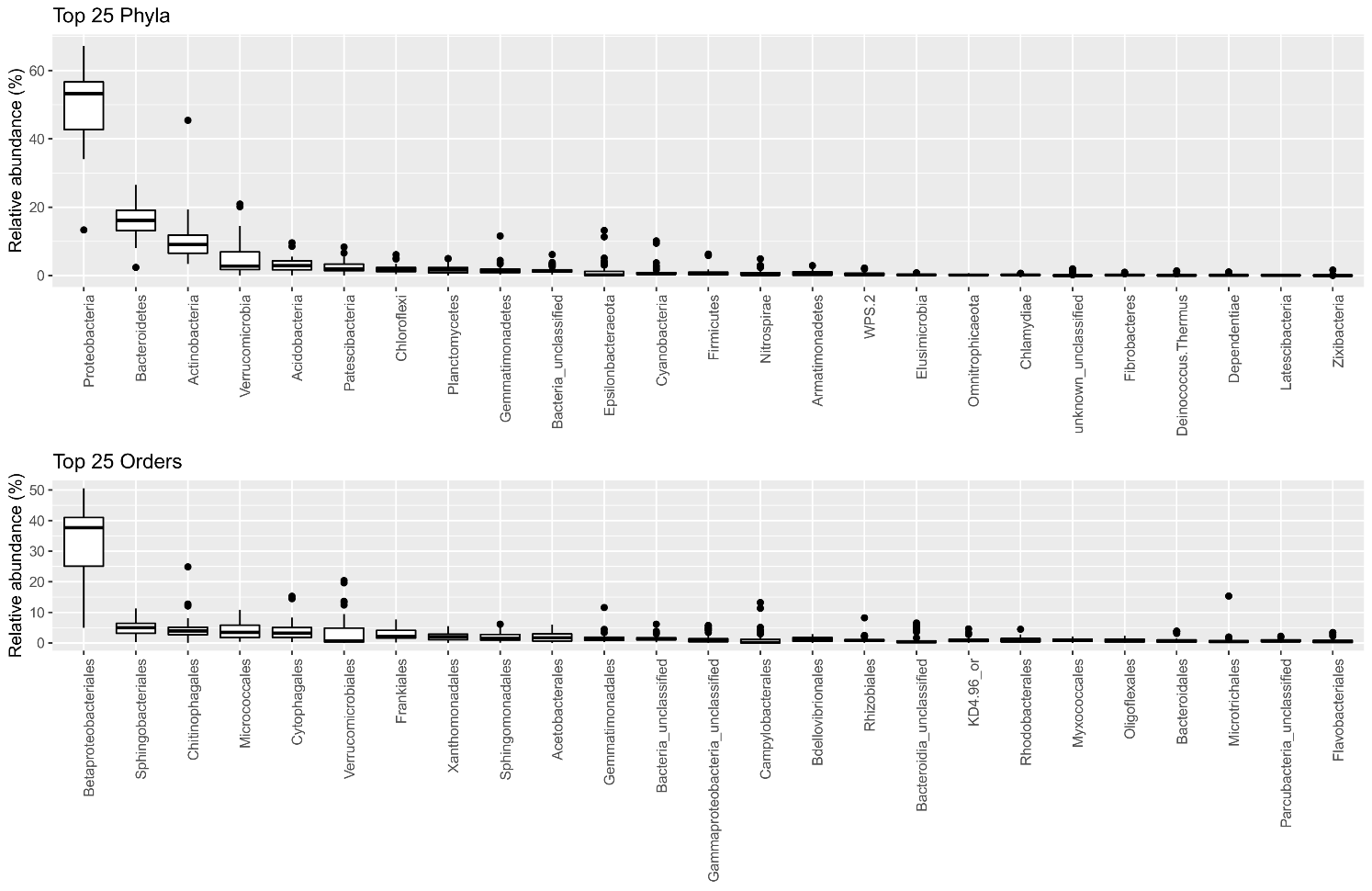


**Supplementary Table 1:** Table of the top 50 OTUs, their inferred habitat and metabolism (see corresponding excel file).
